# 磁性亲水亲脂平衡萃取材料辅助基质固相分散萃取-高效液相色谱-串联质谱法同时测定中药材中76种农药残留

**DOI:** 10.3724/SP.J.1123.2021.08014

**Published:** 2022-04-08

**Authors:** Dan WEI, Ming GUO

**Affiliations:** 1.河北经贸大学生物科学与工程学院, 河北 石家庄 050061; 1. College of Bioscience and Engineering, Hebei University of Economics and Business; Shijiazhuang 050061, China; 2.浙江省化工产品质量检验站有限公司, 浙江 杭州 310023; 2. Zhejiang Chemical Production Quality Inspection Co., Ltd, Hangzhou 310023, China

**Keywords:** 高效液相色谱-串联质谱, 磁性基质固相分散萃取, 磁性亲水亲脂平衡萃取材料, 多农药残留, 中药材, high performance liquid chromatography-tandem mass spectrometry (HPLC-MS/MS), magnetic matrix solid phase dispersion (MMSPD) extraction, magnetic balanced hydrophilic-lipophilic adsorbents, multi-pesticide residues, traditional Chinese medicine (TCM)

## Abstract

建立灵敏、可靠的中药材中农药多残留的检测方法对保证中药材的质量和安全十分重要。制备了磁性亲水亲脂平衡萃取材料Fe_3_O_4_@PLS,将其应用于农药多残留的磁性基质固相分散萃取中,并结合高效液相色谱-串联质谱法(HPLC-MS/MS)检测了金银花、菊花和三七块根(干)3种中药材中76种农药残留量。研究通过扫描电子显微镜(SEM)、傅里叶变换红外光谱(FT-IR)和X-射线衍射仪(XRD)对磁性萃取材料Fe_3_O_4_@PLS进行表面形貌和结构的表征。同时考察了影响磁性基质固相分散萃取效率的主要因素,结果表明,磁性萃取材料Fe_3_O_4_@PLS的用量为10 mg、研磨分散吸附时间为5 min、淋洗液为10 mL 20%(v/v)甲醇水溶液、涡旋振荡清洗1 min、以0.5 mL 0.1%(v/v)甲酸乙腈为洗脱剂、涡旋振荡洗脱1 min, 76种农药的萃取效果最佳。在实际应用中,76种农药在金银花、菊花、三七块根(干)3种中药材中的萃取回收率别为69.1%~112.2%、67.1%~102.8%和70.1%~105.1%,相对标准偏差分别为2.0%~12.4%、2.1%~13.2%和2.0%~13.5%。该方法利用Fe_3_O_4_@PLS良好的磁响应性和亲水亲脂通用吸附特性,可以同时萃取极性农药(如多菌灵等)和非极性农药(如敌瘟磷等),建立了测定中药材中76种农药残留的磁性基质固相分散萃取-高效液相色谱-串联质谱联用的分析方法,具有低消耗、操作简便、灵敏度高等优点,适用于非液态中药材基质中多种类农药残留的检测。

## Abstract (en)

Traditional Chinese medicine (TCM) is gaining popularity worldwide, but its quality is often affected by excessive pesticide residues during cultivation and production. A sensitive and reliable method for the simultaneous determination of multi-residue pesticides in TCMs is the key to guarantee the quality and safety of TCMs. In this study, broad-spectrum hydrophilic-lipophilic balanced magnetic adsorbents were prepared for the magnetic matrix solid phase dispersion (MMSPD) extraction of 76 pesticides from three different TCMs before their detection by high performance liquid chromatography-tandem mass spectrometry (HPLC-MS/MS). Unlike the traditional matrix solid phase dispersion (MSPD), which required tedious SPE column packing, an external magnetic field was employed in our method for magnetic isolation and extraction from TCM samples, followed by grounding adsorption. First, broad-spectrum hydrophilic-lipophilic balanced magnetic adsorbents, Fe_3_O_4_@PLS, were successfully fabricated by the swelling polymerization of divinyl benzene (DVB) and *N*-vinyl pyrrolidone (NVP) on the surface of Fe_3_O_4_ magnetic particles. The prepared materials were systematically characterized by scanning electron microscopy (SEM), Fourier transform infrared spectroscopy (FT-IR), and X-ray diffractometry (XRD) for their morphologies, chemical structure, and crystalline structure. Then, the obtained magnetic particles (Fe_3_O_4_@PLS) were applied to MMSPD for the simultaneous extraction and analysis of the 76 pesticides in honeysuckle, chrysanthemum, and pseudo-ginseng root (dry) prior to detection by HPLC-MS/MS. In order to establish the optimal extraction conditions, the key parameters affecting the MMSPD extraction efficiency were optimized, including the amount of magnetic materials Fe_3_O_4_@PLS (5, 10, 12, 15, 20 mg), volume fractions of methanol in water for matrix purification (0%, 5%, 10%, 20%, 25%), grinding adsorption time for dispersion (2, 3, 4, 5, 6 min), type (methanol, methanol containing 0.1% (v/v) formic acid, acetonitrile, acetonitrile containing 0.1% (v/v) formic acid) and volume (0.5, 1, 2, 2.5, 5 mL) of elution solvent, and vortex time for desorption (1, 2, 3, 4, 5 min). Finally, the optimal conditions were set as follows: 10 mg of the magnetic adsorbent Fe_3_O_4_@PLS, 10 mL 20% (v/v) of methanol in water with a vortex time of 1 min for matrix purification, grinding time of 5 min for dispersion and adsorption, 0.5 mL acetonitrile containing 0.1% (v/v) formic acid as the elution solvent, and vortex time of 1 min for desorption. Then, the 76 pesticides were separated on an Agilent ZORBAX Eclipse Plus C18 column (100 mm×3.0 mm, 1.8 μm) with gradient elution and analyzed in multiple reaction monitoring (MRM) mode by positive electrospray ionization (ESI^+^). Under the optimal conditions, good linearities were obtained for the 76 pesticides in the concentration ranges of 10 to 200 μg/kg, with correlation coefficients (*r* ^2^)≥0.9965. The limits of detection (LODs, *S/N*=3) were in the range of 0.6-3.0 μg/kg, and the limits of quantification (LOQs, *S/N*=10) ranged from 2.0 to 10.0 μg/kg. The proposed method was successfully applied to 76 pesticide residue analysis in honeysuckle, chrysanthemum, and pseudo-ginseng root (dry). At three spiked levels, the recoveries were 69.1%-112.2%, 67.1%-102.8% and 70.1%-105.1%, with RSDs of 2.0%-12.4%, 2.1%-13.2% and 2.0%-13.5% for honeysuckle, chrysanthemum, and pseudo-ginseng root(dry), respectively. The prepared magnetic material Fe_3_O_4_@PLS has the characteristics of both strong magnetic responsibility and high broad-spectrum adsorption property for hydrophilic (e. g., carbendazim) and lipophilic (e. g., edifenphos) pesticides. The implementation of the MMSPD method before the analysis of the 76 pesticides by HPLC-MS/MS has the following advantages: less consumption of the magnetic materials, sample, and organic solvent; simple operation; high sensitivity; and satisfactory accuracy and precision. Hence, this is a potential method for the simultaneous determination of multiple pesticide residues in complex non-liquid TCM samples.

中草药主要来源于天然药及其加工品,其中以植物药居多,是中华文化的传统用药,至今在我国预防治疗、养生与保健中发挥着不可替代的作用^[1]^。随着中草药市场规模的不断扩大,其生产量与消费量不断增加^[2,3]^,大部分野生药材资源已无法满足药用需求,栽培药材已成为目前中药材市场的主流药品。在中药材的栽培过程中不可避免地会发生病虫草害,需要使用农药进行防治,然而农药的频繁使用和滥用会导致中药材中农药残留超标,直接影响我国中药材的质量与安全^[4,5]^。

为了有效控制中药种植中违规使用高安全风险、高残留、高毒农药,从源头把控中草药质量和安全,保障人民用药安全,我国不断改善中草药中农药残留标准。2020年版《中国药典》全面制定植物类中药材和饮片禁用农药的限量标准,不仅有效保障了人民用药的安全性,同时引导中药材种植过程中农药的合理使用,有效控制了大量使用禁用农药和滥用农药等行业共性问题,有助于推进中医药国际化^[6]^。建立可操作性强、准确度高、灵敏度高的中药材中多种农药残留同时筛查和检测的方法显得越来越重要。目前,色谱-质谱联用技术具有高灵敏度、专属性强、高通量检测等优点,能够满足当前快速、准确、高效的检测要求,已广泛用于农药多残留的检测中^[7,8,9]^。中药基质复杂,含有有机酸、糖分、色素等基质干扰物质,需要有效的样品前处理方法实现对样品的萃取富集。目前,应用于农药残留的前处理方法有固相萃取^[10,11]^、液液萃取^[12,13]^和磁性固相萃取^[14]^等。这些方法通常适用于液体样品。对于固体或粉末状中药材样品,需要使用一定量的有机溶剂预提取后再进行净化或吸附萃取。

基质固相分散萃取(MSPD)通过将样品和萃取材料混匀后研磨分散,可以均质并将待测目标物很好地分散至萃取材料表面,以达到良好的分散萃取效果,具有样品使用量少、有机试剂消耗少等优点,适用于固体样品前处理^[15]^。目前,QuEChERS常用于中药材中农药多残留的样品前处理中^[16,17]^。但是,QuEChERS步骤通常包括提取、盐析和离心等,而传统的基质固相分散萃取通常需要采用固相萃取柱进行装柱,操作较为繁琐。同时大多数的吸附剂无法满足农药多残留同时萃取的要求。因此,本实验通过制备具有广谱吸附特性的磁性亲水亲脂平衡萃取材料(Fe_3_O_4_@PLS),可以同时萃取非极性农药和极性农药(log *P<*4.5)^[18]^,将其与粉末状中药材样品一起研磨,在外部磁场的作用下,利用磁性基质固相分散萃取法(MMSPD),实现了中药材中多种类农药的分离和萃取,然后与高效液相色谱-串联质谱法联用,建立了同时测定中药材中76种农药残留的分析方法。

## 1 实验部分

### 1.1 仪器、试剂与材料

Agilent 1290 infinity高效液相色谱-6460 TripleQuad质谱仪、ZORBAX EclipsePlus C18色谱柱(100 mm×3.0 mm, 1.8 μm) (美国Agilent公司); Milli-Q超纯水器(美国Millipore公司);傅里叶变换红外光谱分析仪(FT-IR)(德国Bruker公司); Gemini SEM 500型场发射扫描电子显微镜(SEM) (德国Carl Zeiss公司); D8 Advance型X射线粉末衍射(XRD) (德国Bruker公司)。

甲醇、乙腈为色谱纯,其他试剂均为分析纯;三氯化铁(FeCl_3_·6H_2_O)、正硅酸乙酯(TEOS)、二乙烯基苯(DVB)、*N*-乙烯基吡咯烷酮(NVP)、偶氮二异丁腈(AIBN)、甲基丙烯酸3-(三甲氧基硅基)丙酯(MPS),以及76种农药标准品(10 mg/L)均购自上海安谱实验科技股份有限公司。76种农药标准品(10 mg/L)于-20 ℃冰箱内避光保存,使用前将混合标准储备液恢复至室温,并用甲醇稀释至所需浓度,于4 ℃冰箱内避光保存。中药材样品金银花、菊花、三七块根(干)来源于杭州当地中药材市场、药房。

### 1.2 磁性吸附剂的制备

1.2.1 Fe_3_O_4_纳米颗粒

采用溶剂热法制备^[19]^,准确称取1.9500 g FeCl_3_·6H_2_O分散于22.5 mL乙二醇中,搅拌均匀,加入2.6000 g醋酸钠,搅拌均匀后通入氮气30 min,隔绝空气转移至50 mL反应釜中,于185 ℃条件下反应12 h,静置冷却至室温,使用磁铁从反应溶液中分离得到黑色沉淀,用水、乙醇交替冲洗至近中性,真空烘箱干燥,得到Fe_3_O_4_纳米颗粒。

1.2.2 Fe_3_O_4_@SiO_2_@MPS

参考文献^[20]^方法,称取1.0 g Fe_3_O_4_,置于500 mL三口烧瓶中,加入300.0 mL水,超声分散均匀,加入100 mL乙醇超声分散均匀后,加入1.2 mL纯氨水溶液,涡旋振荡均匀。然后通入氮气,在氮气保护下依次逐滴滴加2.5 mL TEOS和5.5 mL乙醇至上述溶液中,于60 ℃条件下反应12 h,冷却至室温后,利用磁性分离得到黑色沉淀,依次用丙酮和乙醇洗去未反应的TEOS,于50 ℃真空干燥,得到Fe_3_O_4_@SiO_2_纳米颗粒。

称取0.2 g Fe_3_O_4_@SiO_2_,置于500 mL三口烧瓶中,加入30 mL乙醇,超声分散均匀后,用移液枪逐滴滴加3 mL MPS,于25 ℃搅拌24 h,反应完成后,利用磁性分离得到黑色沉淀,用乙醇洗去未反应的MPS,于50 ℃真空干燥,得到Fe_3_O_4_@SiO_2_@MPS。

1.2.3 Fe_3_O_4_@PLS磁性材料

称取3.0 g Fe_3_O_4_@SiO_2_@MPS,置于1000 mL三口烧瓶中,加入500.0 mL乙腈,超声10 min,依次加入6.3 mL DVB、7.5 mL NVP和0.1800 g AIBN,超声分散均匀,将烧瓶中的溶液转移至蒸馏装置中,收集乙腈层,于75 ℃预聚合反应20 min,于115 ℃聚合反应1 h。反应完成后静置冷却至室温,加入收集得到的乙腈溶液(约150.0~200.0 mL),超声分散,然后在外加磁场作用下收集沉淀物,依次用乙腈和乙醇清洗,于50 ℃真空干燥后得到核壳结构的Fe_3_O_4_@PLS。

### 1.3 中药材样品前处理

研磨分散吸附:取50 g中药材,充分粉碎,过3号筛(50目)后,放入聚乙烯袋中备用。准确称取10 mg已粉碎的中药材样品,置于研钵中,加入10 mg磁性吸附剂Fe_3_O_4_@PLS,研磨分散吸附5 min。

淋洗:将研磨均匀的混合物转移至25 mL烧杯中,加入10 mL 20%(v/v)甲醇水溶液,涡旋振荡1 min,利用磁铁磁性分离淋洗液和混合物,收集混合物,弃去淋洗液。

洗脱:在上述收集后的混合物中加入0.5 mL 0.1%(v/v)甲酸乙腈,涡旋振荡1 min进行洗脱,待洗脱完全后,使用磁铁磁性分离,收集洗脱液,经0.22 μm微孔滤膜过滤后进行HPLC-MS/MS定量分析。

### 1.4 HPLC-MS/MS条件

色谱分离采用ZORBAX Eclipse Plus C18色谱柱(100 mm×3.0 mm, 1.8 μm);柱温:40 ℃;流速:0.2 mL/min;流动相:0.05% (v/v)甲酸水溶液(A)和乙腈(B)。梯度洗脱程序:0~4 min, 90%A~50%A; 4~15 min, 50%A~40%A; 15~18 min, 40%A~20%A; 18~19 min, 20%A~90%A; 19~25 min, 90%A。进样量:10 μL。

离子源为电喷雾电离(ESI)源,采用正离子检测模式;多反应监测(MRM)模式进行定量分析。干燥气温度为300 ℃,干燥气流速为5 L/min;雾化气压力为0.3 MPa;鞘气温度:250 ℃,鞘气流速:10 L/min;毛细管电压为4000 V。76种农药的其他质谱参数见表1。

**表1:** 76种农药的质谱参数

No.	Analyte		CAS No.	t_R_/min	Precursor ion (m/z)	Product ion (m/z)	Fragment voltage/V	Collision energy/V
1	cyromazine (灭蝇胺)		66215-27-8	1.364	167.0	85.0	120	25
2	carbendazol (多菌灵)		10605-21-7	2.218	192.1	160.1	80	15
3	atraton (莠去通)		1610-17-9	3.498	212.2	170.2	120	15
4	desmetryn (敌草净)		1014-69-3	4.027	214.1	172.1	120	15
5	prometon (扑灭通)		1610-18-0	4.067	226.2	142.0	120	20
6	terbuthylon (特丁通)		33693-04-8	4.152	226.2	170.1	120	15
7	imazalil (抑霉唑)		35554-44-0	4.785	297.0	159.0	120	20
8	flumazenil (除草定)		314-40-9	4.804	261.0	205.0	80	10
9	cyprazine (环丙津)		22936-86-3	4.915	228.2	186.1	120	15
10	ametryn (莠灭净)		834-12-8	4.915	228.2	186.0	120	20
11	monuron (灭草隆)		150-68-5	4.933	199.0	72.0	120	15
12	dichlorovos (敌敌畏)		62-73-7	5.166	221.0	109.0	120	15
13	carbofuran (克百威)		1563-66-2	5.795	222.3	165.1	120	5
14	chlorotoluron (绿麦隆)		15545-48-9	6.064	213.1	72.0	80	25
15	diuron (敌草隆)		330-54-1	6.256	233.1	72.0	120	20
16	fluometuron (伏草隆)		2164-17-2	6.256	233.1	72.0	120	20
17	terbutryn (特丁净)		886-50-0	6.263	242.2	186.1	120	15
18	phorate-sulfoxide (甲拌磷亚砜)		3-6-2588	6.289	277.0	143.0	100	15
19	rabenzazol (吡咪唑)		40341-04-6	6.316	213.2	172.0	120	25
20	atrazine (莠去津)		1912-24-9	6.333	216.0	174.2	120	15
21	flutriafol (粉唑醇)		76674-21-0	6.362	302.1	70.0	120	15
22	colophonate (噻唑硫磷)		98886-44-3	6.380	284.1	228.1	80	5
23	N,N-diethyl-3-methylbenzamide (避蚊胺)		134-62-3	6.637	192.2	119.0	100	15
24	fenfuram (甲呋酰胺)		58810-48-3	6.640	282.1	160.2	120	20
25	metalaxyl-M (精甲霜灵)		70630-17-0	6.683	280.1	192.1	100	15
26	metalaxyl (甲霜灵)		57837-19-1	6.683	280.1	192.2	120	15
27	azaconazole (戊环唑)		60207-31-0	6.723	300.1	231.1	100	15
28	metobromuron (溴谷隆)		3060-89-7	7.036	259.0	170.1	80	15
29	methyl paraoxon (甲基对氧磷)		311-45-5	7.061	276.2	220.1	100	10
30	dimethametryn (阔草净)		22936-75-0	7.337	256.2	186.1	140	20
31	fensulphothion (丰索磷)		115-90-2	7.357	309.0	157.1	120	25
32	metazachlor (吡唑草胺)		67129-08-2	7.475	278.1	134.1	80	20
33	cotofor (杀草净)		4147-51-7	7.479	256.1	144.1	140	30
34	diphenamid (双苯酰草胺)		957-51-7	7.871	240.1	134.1	120	20
35	propanil (敌稗)		709-98-8	8.042	218.0	162.1	120	15
36	dichlofenthion (虫线磷)		297-97-2	8.045	249.1	97.0	80	30
37	propazine (扑灭津)		139-40-2	8.099	229.9	146.1	120	20
38	triadimenol (三唑醇)		55219-65-3	8.471	296.1	70.0	80	10
39	paclobutrazol (多效唑)		76738-62-0	8.623	294.2	70.0	100	15
40	terbuthylazine (特丁津)		5915-41-3	8.761	230.1	174.1	120	15
41	fenobucarb (仲丁威)		3766-81-2	8.812	208.2	95.0	80	10
42	lorox (利谷隆)		330-55-2	9.127	249.0	160.1	100	15
43	phenmedipham (甜菜宁)		13684-63-4	9.242	301.1	168.1	80	5
44	tiancaian (甜菜胺)		13684-56-5	9.276	301.2	182.1	80	5
45	flusilazole (氟硅唑)		85509-19-9	10.462	321.1	119.0	100	25
46	pyriftalid (环酯草醚)		135186-78-6	10.521	319.0	139.1	140	35
47	cyproconazole (环丙唑醇)		94361-06-5	10.541	292.1	70.0	120	15
48	uniconazole (烯效唑)		83657-22-1	10.541	292.1	70.1	120	30
49	myclobutanil (腈菌唑)		88671-89-0	10.542	289.1	125.0	120	20
50	ruelene (育畜磷)		299-86-5	10.717	292.1	236.0	120	20
51	ethoprophos (灭线磷)		13194-48-4	10.861	243.1	173.0	120	10
52	pyridaphethione (哒嗪硫磷)		119-12-0	11.608	341.1	189.2	120	20
No.		Analyte	CAS No.	t_R_/min	Precursor ion (m/z)	Product ion (m/z)	Fragment voltage/V	Collision energy/V
53		terbufos sulfone (特丁磷砜)	56070-16-7	11.612	321.2	171.1	80	5
54		pretilachlor (丙草胺)	51218-49-6	12.289	316.1	247.1	120	15
55		penconazole (戊菌唑)	66246-88-6	12.334	284.1	70.0	120	15
56		iprobenfos (异稻瘟净)	26087-47-8	12.342	289.1	91.0	80	25
57		alachlor (甲草胺)	15972-60-8	12.494	270.2	238.2	80	10
58		isazofos (氯唑磷)	42509-80-8	12.752	314.1	162.1	100	10
59		flutolanil (氟酰胺)	66332-96-5	12.903	324.2	262.1	30	25
60		diniconazole (烯唑醇)	83657-24-3	12.968	326.1	70.0	120	25
61		azinphos (嘧硫磷)	5221-49-8	13.147	306.1	170.2	120	20
62		buprofezin (噻嗪酮)	69327-76-0	13.347	306.2	201.0	120	15
63		diazinon (二嗪磷)	333-41-5	13.470	311.1	283.0	100	10
64		mecarbam (灭蚜磷)	2595-54-2	13.727	330.0	227.0	80	5
65		tebufenozide (虫酰肼)	112410-23-8	13.942	297.0	133.0	80	15
66		pirimiphos-ethyl (乙嘧硫磷)	38260-54-7	14.569	293.1	125.0	80	20
67		pirimiphos-methyl (甲基嘧啶磷)	29232-93-7	14.592	306.2	164.0	120	20
68		cadusafos (硫线磷)	95465-99-9	14.621	271.1	159.1	80	10
69		triallate (野麦畏)	2303-17-5	14.860	305.0	169.1	160	20
70		fonofos (地虫硫磷)	944-22-9	15.240	247.1	109.0	80	15
71		vernolate (灭草敌)	1929-77-7	15.291	204.2	128.2	100	10
72		dehydro barnidipine (治螟磷)	3689-24-5	15.765	323.0	171.1	120	10
73		ediphenphos (敌瘟磷)	17109-49-8	16.185	312.1	252.1	100	15
74		pyrimitate (嘧啶磷)	23505-41-1	17.139	334.2	198.2	120	20
75		S-bioallethrin (烯丙菊酯)	584-79-2	17.284	303.2	135.1	60	10
76		tebupirimfos (丁基嘧啶磷)	96182-53-5	17.425	319.1	277.1	120	10

## 2 结果与讨论

### 2.1 磁性萃取材料的表征

Fe_3_O_4_@PLS和Fe_3_O_4_纳米颗粒的粒径大小和表面形态如图1a所示,Fe_3_O_4_纳米颗粒表面较为光滑,呈规则的球形,颗粒形状大小较为均匀,粒径约为50 nm。通过硅烷化、双键以及有机共聚物修饰后得到的Fe_3_O_4_@PLS粒径约为500~600 nm,呈较规则的球形,粒径远大于Fe_3_O_4_纳米颗粒,且其表面有大量褶皱,增大了比表面积,提高了对目标物的吸附效率。如图1b所示,30.12°、35.47°、43.11°、53.49°、57.06°和62.62°的2*θ*值的特征峰对应于Fe_3_O_4_的(222)、(331)、(400)和(422)、(511)和(440), PLS无定形结构的特征尖峰、Fe_3_O_4_@PLS的特征峰与Fe_3_O_4_特征峰一致。

**图1:**
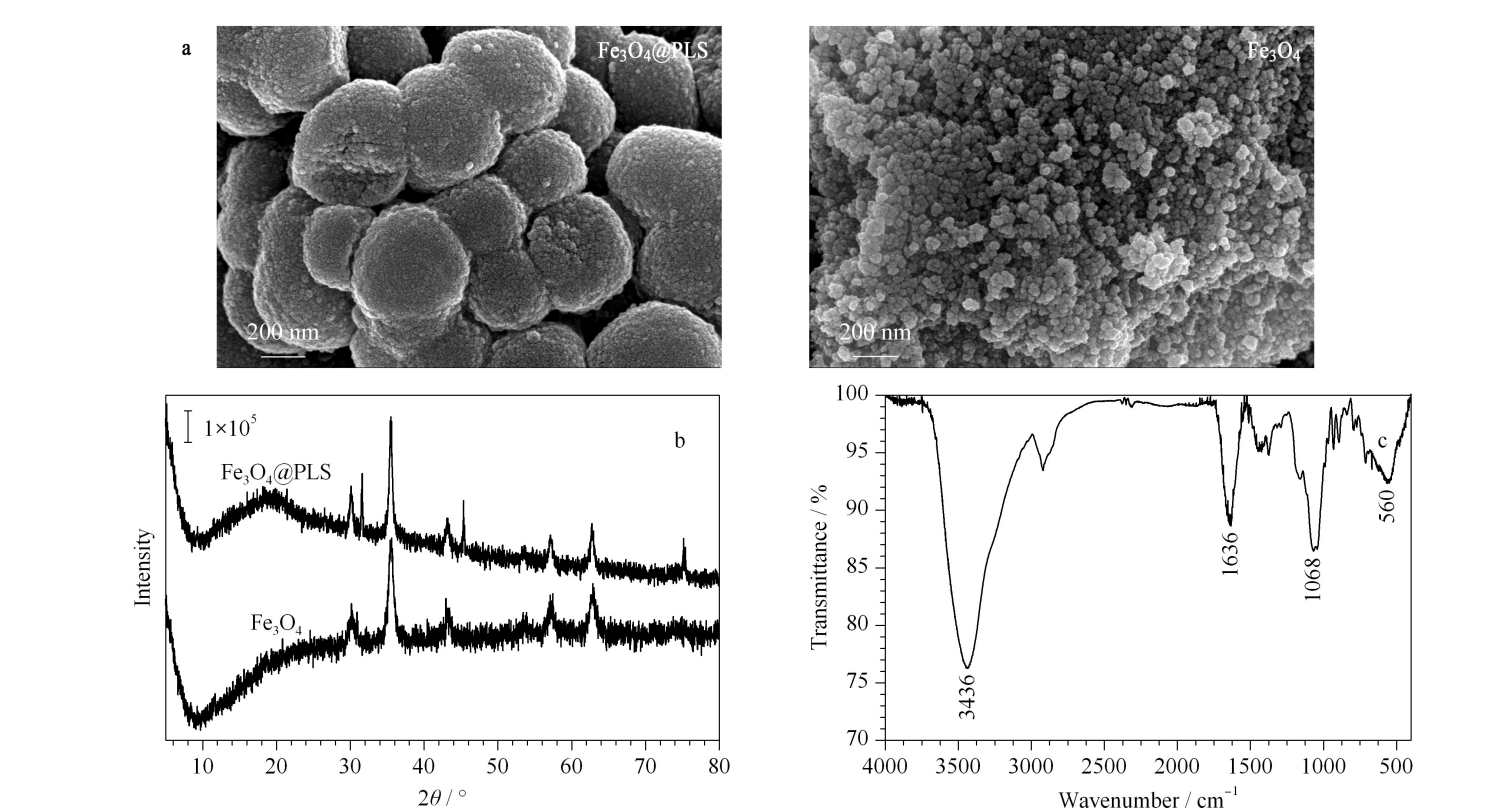
Fe_3_O_4_@PLS和Fe_3_O_4_的表征结果

此外采用傅里叶变换红外光谱(FT-IR)对Fe_3_O_4_@PLS进行表征,如图1c所示,560 cm^-1^处为Fe_3_O_4_中Fe-O-Fe的伸缩振动特征峰,1068 cm^-1^处为Si-O-Si基团的面内伸缩振动的特征吸收峰,1636 cm^-1^、3436 cm^-1^处分别为Si-OH的弯曲振动峰和伸缩振动峰,说明Fe_3_O_4_表面形成SiO_2_涂层,而得到Fe_3_O_4_@SiO_2_,在1500~1600 cm^-1^处为DVB中芳环的C=C弯曲振动特征吸收峰,1250~1300 cm^-1^和1400~1500 cm^-1^处分别为NVP中C-C伸缩振动特征峰和C-N伸缩振动特征峰^[21]^,综上所述,表明DVB和NVP单体成功负载于Fe_3_O_4_表面。

### 2.2 MMSPD萃取条件的优化

2.2.1 磁性萃取剂用量

磁性萃取剂用量是影响萃取效率的重要因素之一。实验考察了Fe_3_O_4_@PLS的用量(5、10、12、15、20 mg)对76种农药萃取回收率的影响。如图2a所示,Fe_3_O_4_@PLS用量为5~10 mg时,多数目标物的回收率随着磁性吸附剂用量的增加而增加,当Fe_3_O_4_@PLS用量为10 mg时,75种农药萃取回收率≥70%,其中,62种农药的萃取回收率为80%~120%,继续增加Fe_3_O_4_@PLS用量,回收率<70%的目标物个数呈增加趋势。因此选择Fe_3_O_4_@PLS的用量为10 mg。

**图2:**
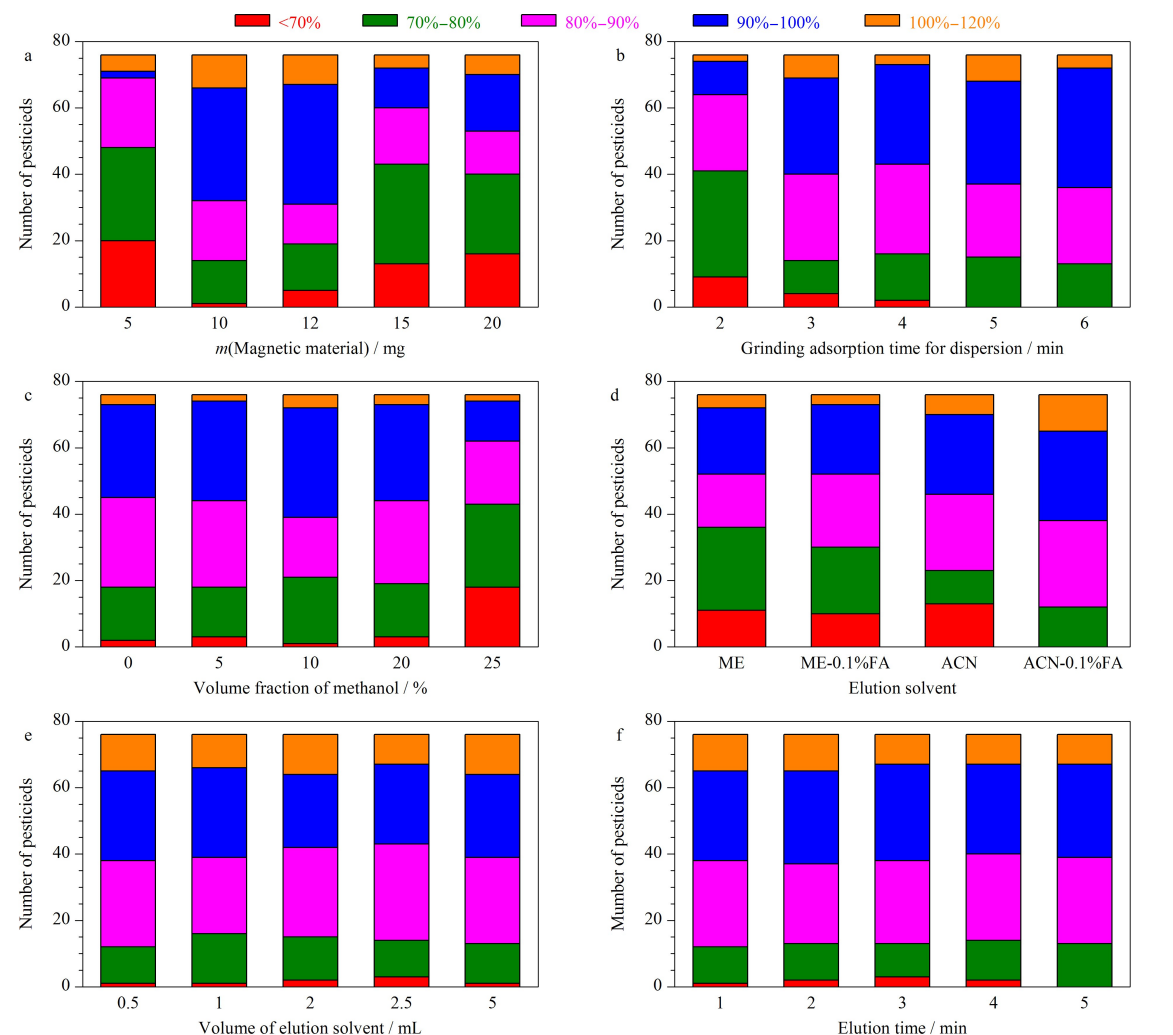
不同萃取条件对76种农药回收率的影响

2.2.2 研磨分散吸附时间

磁性吸附剂在样品中的分散程度是影响MMSPD萃取回收率的关键因素之一,充足的研磨分散时间可以使样品基质中的目标物充分吸附于磁性吸附剂表面,进而提高萃取回收率。实验对比了不同研磨分散时间(2、3、4、5、6 min)的萃取回收率。如图2b所示,当研磨时间从2 min增加至5 min时,回收率≥70%的目标物个数明显增加,研磨时间为5 min时,76种农药萃取回收率≥70%,继续增加研磨分散时间,回收率≥70%和回收率在80%~100%的目标物个数变化均不显著。因此最终确定研磨分散时间为5 min。

2.2.3 淋洗液中甲醇的体积分数

磁性萃取剂和样品混合物研磨分散后使用甲醇水溶液作为淋洗液。甲醇水溶液通常可以作为金银花、菊花和三七有效成分的提取剂^[22,23,24]^,适当增加淋洗液中甲醇的体积分数有利于去除金银花、菊花和三七中的基质干扰,同时淋洗液中甲醇的体积分数也会影响磁性萃取剂对目标物的萃取效率。实验对比了甲醇以及体积分数为5%、10%、20%、25%的甲醇水溶液对76种农药回收率的影响。如图2c所示,当甲醇体积分数<20%时,约73~75种目标物萃取回收率大于70%,继续增加有机相的体积分数,回收率<70%的目标物个数明显增加。因此,为了获得更好的回收率且能去除更多的杂质,选择20%甲醇水溶液作为淋洗液。

2.2.4 洗脱剂种类

采用涡旋振荡对目标物进行洗脱,以甲醇、0.1%甲酸甲醇、乙腈、0.1%甲酸乙腈作为洗脱剂进行考察。以乙腈作为洗脱剂时,3种样品的洗脱液较为澄清,说明有机杂质共萃取物较少。如图2d所示,使用乙腈、甲醇作为洗脱剂时,萃取回收率为80%~120%的目标物个数分别为43和40,说明乙腈的萃取回收率相对优于甲醇。乙腈中加入0.1%甲酸后,76种农药的萃取回收率均≥70%,其中,64种农药的萃取回收率达到80%~120%。因此最终选择0.1%甲酸乙腈溶液作为洗脱剂。

2.2.5 洗脱剂体积

实验对比了洗脱剂体积分别为0.5、1、2、2.5和5 mL时76种农药的萃取回收率。如图2e所示,洗脱剂体积为0.5 mL时,除地虫硫磷的萃取回收率为68.56%外,其他75种农药的回收率≥70%。因此选择0.5 mL作为洗脱剂体积。

2.2.6 洗脱时间

实验同时考察了涡旋振荡洗脱时间为1、2、3、4和5 min时76种农药萃取回收率的变化。如图2f所示,当涡旋洗脱时间为1 min时,除敌敌畏的回收率为65.12%外,75种目标物的萃取回收率均大于70%,继续增加涡旋时间,目标物的萃取回收率未有显著变化,为了保证检测效率,选择涡旋振荡洗脱时间为1 min。

### 2.3 方法验证

2.3.1 基质效应

基质效应是由于样品中基质与待测目标物共存而导致待测目标物在仪器中响应信号有不同程度增强或抑制的现象,从而影响测定的精确度。为了考察基质效应,选择3种空白基质萃取液,配制系列空白基质匹配标准溶液(0.5、1.0、10、50和100 μg/L),同时用甲醇逐级稀释配制相同浓度的溶剂混合标准溶液。通过计算基质匹配标准曲线与溶剂混合标准曲线斜率的比值来考察基质效应。若等于1,表明无基质效应;若比值范围为0.8~1.2,表明无明显基质效应或基质效应可以忽略。结果表明,金银花、菊花、三七块根(干)3种中药材的基质效应值分别为0.58~1.30、0.81~1.45和0.73~1.36,均超出上述无明显基质效应范围,3种中药材的基质效应主要表现为基质增强效应。因此本实验采用空白基质匹配标准补偿基质效应。

2.3.2 线性范围、检出限和定量限

用1.3节方法处理后的空白中药材萃取液稀释混合农药标准储备液,配制成2、10、20、100和200 μg/kg的系列基质匹配标准溶液,在优化实验条件下进行测定,得到线性方程和线性相关系数(*r*^2^), 76种农药在10~200 μg/kg范围内,呈良好的线性关系,相关系数均大于0.9965。以*S/N*≥3和*S/N*≥10分别计算检出限(LOD)、定量限(LOQ),结果见表S1所示(详见http://www.chrom-China.com)。

2.3.3 回收率和精密度

采用1.3节样品处理方法,对金银花、菊花、三七块根(干)3种中药材进行76种农药3个水平(20、80和150 μg/kg)的加标回收率试验,每个水平重复3次,采用外标法定量。如表S2所示,3个水平下,金银花、菊花、三七块根(干)3种中药材的萃取回收率分别为69.1%~112.2%、67.1%~102.8%和70.1%~105.1%, RSD分别为2.0%~12.4%、2.1%~13.2%和2.0%~13.5%, 满足药用植物检测要求^[25]^,说明所建立方法整体准确度和精密度良好。

### 2.4 方法比较

本文所建立方法与其他文献中报道方法比较如表2所示,本文所使用方法具有低消耗(样品、磁性萃取材料用量)、操作简便、灵敏度较高等优点,适用于非液态中药材基质中多种类农药残留的检测。

**表2:** 本方法与其他方法的比较

Sample	Method	Adsorbent	Analytes	Amount of sample	Amount of adsorbent		LOQ		Ref.
Water	SPE-HPLC-MS/MS	Cleanrt^®^-PEP SPE column	18 pesticides	300 mL	500	mg	10	ng/L	[10]
Bayberry	Pass-through SPE-	PRiME HLB SPE column	29 pesticides	5 g	200	mg	6.0	μg/kg	[11]
	UPLC-MS/MS								
Commercial	DLLME-UPLC-MS/MS	PSA	6 pesticides	4 g	1.5	g	0.06-0.10	μg/kg	[13]
herbal tea									
Herb tea	QuECHERS-HPLC-MS/MS	C_18_+PSA+GCB	77 pesticides	2 g	1.05	g	3.3-33.3	μg/kg	[14]
TCMs	MMSPD-HPLC-MS/MS	Fe_3_O_4_@PLS	76 pesticides	10 mg	10	mg	1.7-20.7	μg/kg	this work

TCMs: traditional Chinese medicine; DLLME: dispersive liquid-liquid microextraction; MMSPD: magnetic matrix solid phase dispesion; PEP: polar enhanced polymer; HLB: hydrophile-lipophile balance.

### 2.5 实际样品测定

将建立的MMSPD-HPLC-MS/MS分析方法应用于3种中药材,包含市售金银花5个、菊花3个、三七块根(干)2个共计10个样品,除1个金银花检出多菌灵外,其余样品均未检出农药残留或低于定量限,并且检出阳性金银花样品中多菌灵的残留量未超出2020年版中《中国药典》的限度规定^[26]^。金银花阳性样品的多反应监测色谱图见图3。

**图3:**
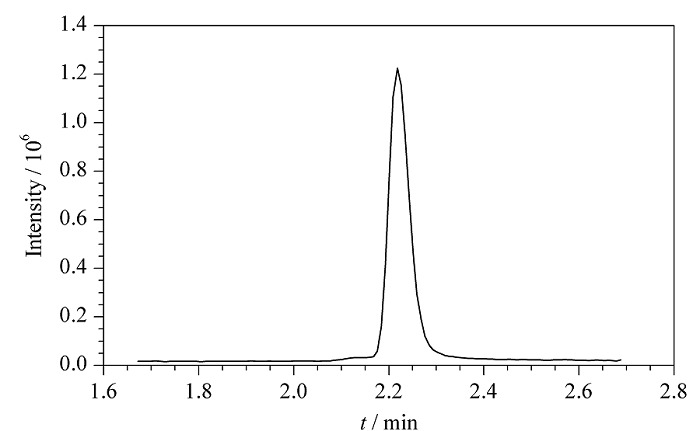
金银花阳性样品的多反应监测色谱图

同时,采用GB 23200.11-2016和本文所建立方法对金银花阳性样品进行定量分析,平行测定5次,以GB 23200.11-2016方法所测得多菌灵的平均含量(11.9 μg/kg)为已知值,本文所建立方法多菌灵的5次检出含量依次为11.6、12.7、12.9、12.1和13.2 μg/kg,采用*t*检验法比较本方法测定值和已知值,*t*_测定_=2.20<*t*_(0.95, _*_n_*_=5)_=2.57,说明本文所建立方法与GB 23200.11-2016相比,检测结果基本一致,无显著差异,具有一定的准确性和可靠性。

## 3 结论

本研究针对中药材农药多残留检测中前处理步骤复杂、吸附剂萃取目标物种类少等问题,制备了磁性亲水亲脂平衡材料Fe_3_O_4_@PLS,采用磁性萃取剂与样品直接研磨分散,无需有机溶剂预提取,同时借助施加外部磁场进行磁性基质固相分散萃取,有效简化了传统基质分散固相萃取的装柱步骤,结合高效液相色谱-串联质谱法进行检测,建立了一种有效测定固体或粉末状中药材样品中农药多残留的方法。本方法在非液体中药材中痕量农药多残留的检测方面具有较高的应用价值,将为中药材中农药残留的筛查和质量控制的研究提供可靠依据。
